# Structural OCT and OCT angiography biomarkers associated with the development and progression of geographic atrophy in AMD

**DOI:** 10.1007/s00417-024-06497-8

**Published:** 2024-04-30

**Authors:** Veronica Vallino, Alessandro Berni, Andrea Coletto, Sonia Serafino, Francesco Bandello, Michele Reibaldi, Enrico Borrelli

**Affiliations:** 1 Department of Surgical Sciences, University of Turin, Corso Dogliotti 14, Turin, Italy; 2 Department of Ophthalmology, “City of Health and Science” Hospital, Turin, Italy; 3 Vita-Salute San Raffaele University Milan, Milan, Italy; 4 IRCCS San Raffaele Scientific Institute, Milan, Italy

**Keywords:** Age-related macular degeneration, Optical coherence tomography, geographic atrophy, Biomarkers, Drusen, Reticular pseudodrusen

## Abstract

**Background:**

Geographic atrophy (GA) is an advanced, irreversible, and progressive form of age-related macular degeneration (AMD). Structural optical coherence tomography (OCT) and OCT angiography (OCTA) have been largely used to characterize this stage of AMD and, more importantly, to define biomarkers associated with the development and progression of GA in AMD.

**Methods:**

Articles pertaining to OCT and OCTA biomarkers related to the development and progression of GA with relevant key words were used to search in PubMed, Researchgate, and Google Scholar. The articles were selected based on their relevance, reliability, publication year, published journal, and accessibility.

**Results:**

Previous reports have highlighted various OCT and OCTA biomarkers linked to the onset and advancement of GA. These biomarkers encompass characteristics such as the size, volume, and subtype of drusen, the presence of hyperreflective foci, basal laminar deposits, incomplete retinal pigment epithelium and outer retinal atrophy (iRORA), persistent choroidal hypertransmission defects, and the existence of subretinal drusenoid deposits (also referred to as reticular pseudodrusen). Moreover, biomarkers associated with the progression of GA include thinning of the outer retina, photoreceptor degradation, the distance between retinal pigment epithelium and Bruch’s membrane, and choriocapillaris loss.

**Conclusion:**

The advent of novel treatment strategies for GA underscores the heightened need for prompt diagnosis and precise monitoring of individuals with this condition. The utilization of structural OCT and OCTA becomes essential for identifying distinct biomarkers associated with the initiation and progression of GA.


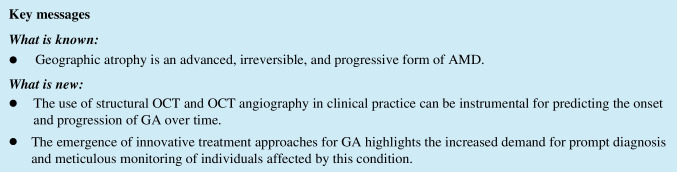


## Introduction

Geographic atrophy (GA), along with macular neovascularization (MNV), represents the late stage of age-related macular degeneration (AMD), the leading cause of legal blindness among the elderly worldwide [1]. In the United States, GA affects approximately 1 million people, with 160,000 new cases annually. The average age of affected people is 79 years, and the cases are expected to increase in the coming years due to the aging population [2]. Gass first described GA in 1970 as "geographic areas of atrophy in the setting of senile macular choroidal degeneration" [3]. However, it was not until 1995 that the International Age-Related Maculopathy Epidemiological Study Group defined the disease and categorized the associated lesions [4].

GA is an advanced, irreversible, and progressive form of AMD, characterized by a gradual loss of retinal pigment epithelium (RPE) cells that results in the formation of macular atrophic lesions. These lesions have well-defined borders and can be seen as contiguous areas of reduced or absent pigmentation on color fundus photography (CFP). Within GA regions, the neuroretinal layers are thinner, the underlying choroid is attenuated or atrophied, and the choroidal vessels become more apparent due to the degeneration of the overlying RPE [5].

In the early stages of GA, central vision is often preserved. However, as the disease progresses at an approximate rate of 1.79 mm [2] per year, there is a significant decline in visual acuity and reading performance, especially once the fovea becomes involved [6–9].

Complement-inhibition therapies have recently been approved by the FDA to help reduce vision loss associated with GA [10]. Although these therapies can slow GA enlargement, they cannot stop disease progression or reverse the vision loss that has already occurred. As such, it is crucial to promptly identify GA patients who would benefit the most from the treatment. Similarly, there is an urgent need to select intermediate AMD (iAMD) patients with a higher risk of GA development for their inclusion in forthcoming interventional studies. Therefore, it appears of great interest to define risk factors associated with the progression from iAMD to GA and the subsequent GA enlargement.

This review aims to emphasize the most crucial biomarkers associated with the onset (i.e., from the early/intermediate AMD stage) and growth of GA. The focus is on enhancing the comprehension of critical optical coherence tomography (OCT) and OCT angiography (OCTA) findings that require sustained attention.

## Retinal imaging in geographic atrophy

Traditionally, CFP has been considered the gold standard imaging technique for the diagnosis and follow-up of GA, while fundus autofluorescence (FAF) has gained prominence due to its high-contrast images. However, FAF is not without limitations as this imaging modality may be challenging to acquire, has limited availability in clinics, and may cause discomfort for patients during examination. More importantly, blue FAF has limitations in the assessment of the foveal region, and borders may be poorly defined due to residual autofluorescent debris present within the atrophy bed [11–13].

Therefore, structural OCT has emerged as the preferred imaging method for patients with AMD. This imaging technique is widely utilized across various clinical settings, facilitating its application in the management of AMD, a prevalent disease. Additionally, OCT enables the capture of high-definition cross-sectional images of the macula, allowing for detailed quantitative analysis of each retinal layer's involvement and loss. Moreover, OCT possesses the ability to detect atrophic processes in their early stages, often before they become clinically apparent through other imaging methods. Lastly, unlike alternative imaging modalities (i.e., blue FAF), OCT excels in assessing the foveal region and can easily identify the presence of RPE atrophy in this crucial area.

**Fig. 1:**
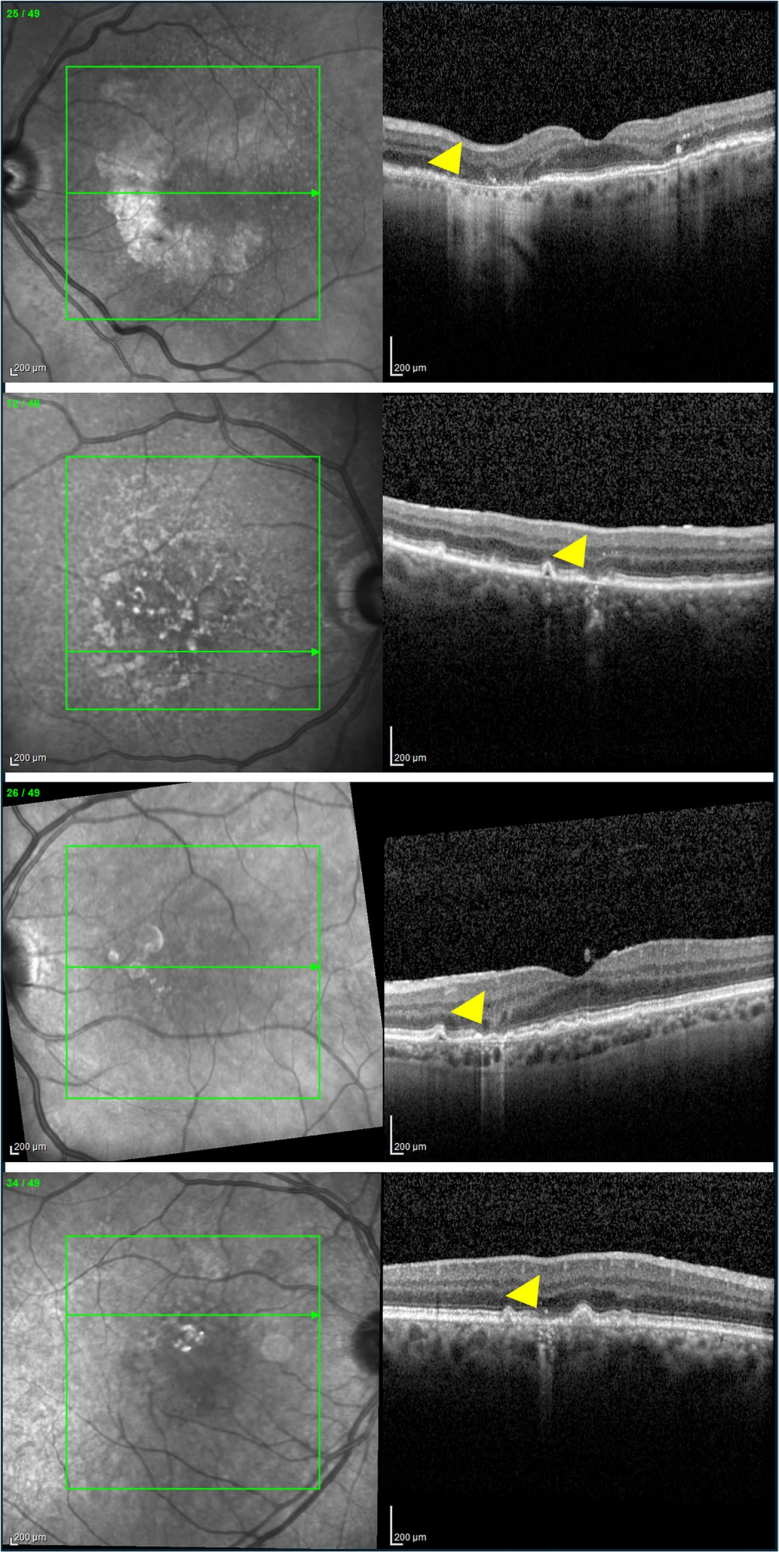
Examples of the OCT-based findings associated with atrophy in AMD as defined by the Classification of Atrophy Meetings (CAM) program [5]. Using structural OCT, the following characteristics may be identified: complete RPE and outer retinal atrophy (cRORA – top row), incomplete RPE and outer retinal atrophy (iRORA – top-middle row), complete outer retinal atrophy (cORA – bottom-middle row), and incomplete outer retinal atrophy (iORA – bottom row). These findings are highlighted using yellow arrowheads

Assuming the importance of OCT in identifying and characterizing GA and the necessity for standardized terminology across trials and clinical practices, the Classification of Atrophy Meetings (CAM) program in 2017 established consensus terminology and OCT-based criteria for defining atrophy in AMD contexts [5]. In detail, the following terms were introduced (Fig. 1):

- Complete RPE and outer retinal atrophy (cRORA): this OCT sign is defined by a region of hypertransmission of at least 250 μm in diameter, and a zone of attenuation or disruption of the RPE of at least 250 μm in diameter, and evidence of overlying photoreceptor degeneration, all occurring in the absence of signs of an RPE tear.
- Incomplete RPE and outer retinal atrophy (iRORA): this OCT feature is characterized by a region of signal hypertransmission into the choroid with a corresponding zone of attenuation or disruption of the RPE, and evidence of overlying photoreceptor degeneration. These OCT findings are typically smaller than 250 μm in iRORA.
- Complete outer retinal atrophy (cORA): is an area identified on OCT imaging by the absence of the EZ and the interdigitation zone (IZ), significant thinning of the outer retina and intermittent choroidal hypertransmission, in the context of an intact RPE band.
- Incomplete outer retinal atrophy (iORA): is defined on OCT by the presence of a zone of continuous ELM and detectable but interrupted EZ in the setting of noticeable thinning of the outer retina with no choroidal hypertransmission and an intact RPE band.

It is worth noting that according to CAM, the term cRORA includes both forms of atrophy associated with or without MNV. However, only the latter subset is specifically termed geographic atrophy [5].

Recently, OCT angiography (OCTA) has also been introduced into the study of GA due to the ease of visualization of the choriocapillaris (CC) as this appears to play a key role in the pathogenesis of the disease [14–25]. In particular, the impairment of CC blood flow is associated with the rate of GA progression: indeed, it has been shown that the density of CC vessels is most reduced along the margins of the lesion [26–31].

## Risk factors for the development of GA

### Drusen

**Fig. 2:**
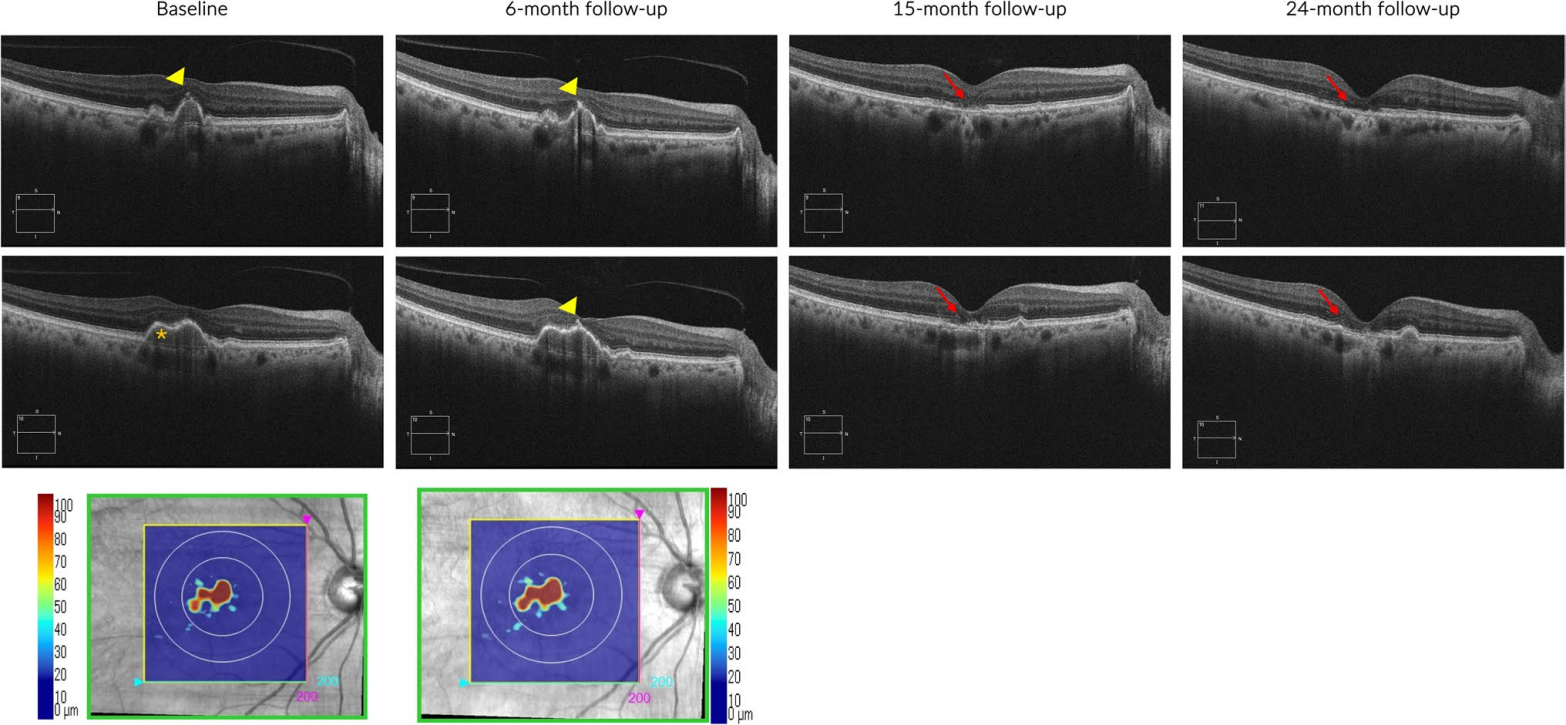
Structural OCT images from a patient with intermediate AMD developing GA. Baseline and 6-month follow-up structural OCT images revealed imaging biomarkers indicative of GA development. These biomarkers included hyperreflective foci (indicated by a yellow arrowhead) and a substantial drusen volume (denoted by an orange asterisk). The Cirrus 6000 OCT device (Zeiss, Dublin, CA) automatically measured the drusen map volume (bottom line), highlighting a noticeable growth in drusen volume over the 6-month period (from 0.12 mm^3^ to 0.15 mm^3^) in the 3-mm central circle. By the 15-month follow-up, the emergence of cRORA became evident, with a further enlargement of the atrophic area observed at the 24-month follow-up visit

Drusen, which consist of extracellular accumulations of debris including lipids, carbohydrates, zinc, and proteins, typically accumulate between the RPE’s basal lamina and Bruch's membrane and represent the hallmark of AMD since its early stages [32]. When employing structural OCT in early/intermediate AMD patients, it becomes crucial to measure the volume of drusen and evaluate the imaging biomarkers that depict the structure of these drusen (Fig. 2). This assessment holds significance as these factors have been linked to the progression from early/intermediate AMD to GA.

The Age-Related Eye Disease Study (AREDS) investigators had shown that drusen may be characterized by a peculiar pattern: the formation of large drusen is followed by their reduction, eventually leading to the development of GA [33]. This process may be occasionally preceded by the appearance of refractile deposits [34]. In details, Cukras et al. [33] analyzed CFP images obtained from the AREDS participants and demonstrated that large drusen (i.e., diameter > 125 μm) and drusenoid pigment epithelial detachments (dPEDs – diameter > 350 μm) are associated with an increased risk of AMD progression. Specifically, their research revealed that among the cases with dPEDs examined, 19% progressed to GA, while 23% developed into MNV.

Structural OCT has notably enhanced our capacity to explore and accurately measure drusen volume (i.e., also known as RPE to Bruch’s membrane volume). This progress is owed to the development of various algorithms specifically designed to automatically segment drusen volume, allowing for precise quantification [35]. Abdelfattah and colleagues [36] first employed structural OCT to investigate whether a role in the progression to late AMD among individuals with early/intermediate AMD. Their findings revealed that patients exhibiting a drusen volume exceeding 0.03 mm^3^ had more than a fourfold higher likelihood of advancing to late AMD compared to those with lower drusen volumes. Successively, Hirabayashi et al. [37] specifically employed structural OCT to determine the frequency of multiple biomarkers of intermediate AMD and their relationship with the development of cRORA after 2 years. The latter study involved 330 eyes of 330 consecutive patients with intermediate AMD in at least 1 eye, all of whom had 24 months of follow-up data. Their findings indicated that drusen volume poses a risk for cRORA onset within a 2-year period.

Recently, Liu et al. [38] longitudinally studied 171 iAMD eyes to design a clinical trial for studying therapies aimed at slowing the progression from iAMD to GA. Using persistent choroidal hypertransmission defects (hyperTDs) as the clinical trial endpoint, the authors found that a drusen volume higher than 0.25 mm^3^ within the 5-mm fovea-centered circle was able to predict that approximately 68% of iAMD eyes would develop a hyperTD within 12 months. Drusen volume, therefore, represents an important risk factor for disease progression, although it must be noted that changes in drusen volume are dynamic and not regularly progressive throughout the course of the disease [37].

The utilization of structural OCT also allows for the examination of reflectivity features within the drusen structure, which could hold significance in defining the likelihood of progression toward GA. Structural OCT enables the identification of crucial features that include: (i) small hyperreflective dots within the drusen; (ii) heterogeneous internal reflectivity within drusen (HIRD); (iii) hyper-reflective lines near Bruch’s membrane [39].

Nassisi et al. [40] have recently evaluated the relationship between OCT features and progression to cRORA in the fellow eyes with intermediate AMD of patients enrolled in the HARBOR study. Among the identified OCT risk factors, hyporeflective foci within drusenoid lesions were consistent risk factors for the development of cRORA. Other risk factors included intraretinal hyperreflective foci and drusen volume.

It is believed that the existence of hyporeflective foci within drusenoid lesions might increase the likelihood of these drusen collapsing due to a weakened structural integrity [41]. Hence, this particular subset of drusen could signify drusen that are in the process of collapsing or undergoing structural breakdown. These pyramidal or dome-shaped hyperreflective lesions were also termed “ghost drusen” [42] or “refractile drusen” [43] and are also characterized by a glistening appearance on CFP which was associated with to the presence of calcium-containing spherules [39]. The latter characteristic has suggested that refractile (i.e., or ghost) drusen may also represent the imaging surrogate of calcified drusen once they undergo RPE atrophy. However, these lesions cannot be considered as a precursor of GA, as these lesions are already associated with RPE atrophy and are commonly identified in GA areas.

Calcified drusen may be visualized using structural OCT as lesions with a hyperreflective cap along with a heterogenous internal reflectivity or hyporeflective core on OCT B-scans. In the AREDS study, drusen categorized as "calcified” displayed on CFP images a glistering appearance with poorly reflective but translucent internal material, because of the presence of calcium inside [34]. The Classification of Atrophy Meetings group have recognized calcified drusen as clinically significant lesions that have a high association with GA (i.e., cRORA) developing [5, 44–47].

In a recent study, Liu et al. [48] have employed multimodal imaging, including structural OCT, to detect and monitor calcified drusen [49]. The study involved 220 eyes from 139 patients with nonexudative AMD, where 42.7% of eyes showed calcified drusen either initially or during follow-up. On *en face* swept-source OCT (SS-OCT) images, calcified drusen appeared as dark focal lesions (i.e., choroidal hypotransmission defects—hypoTDs) detected in the choroid using a sub-RPE slab (located 64–400 µm beneath Bruch’s membrane). Corresponding B-scans revealed drusen with varying internal reflectivity, hyporeflective cores, and hyperreflective caps. Over time, many calcified drusen exhibited choroidal hyperTDs forming around the edges of the hypoTDs, creating a donut-like appearance on *en face* SS OCT images. These donut lesions were linked to notable thinning of the overlying retina, evident as hypoautofluorescence in the corresponding FAF images. These lesions met the criteria for persistent hyperTD, indicating cRORA. Six eyes displayed regression of calcified drusen without developing cRORA. In these cases, B-scans exhibited deposits along the RPE, with thinning of the outer retina in areas previously occupied by calcified lesions. Therefore, the presence of calcified drusen may be considered a recognized risk factor for the development of GA.

### Subretinal drusenoid deposits

Reticular pseudodrusen (RPD) were first described by Mimoun et al. in 1990 as "pseudo-drusen visible en lumiére bleue" [50]. They were successively renamed "reticular drusen" by Klein et al. [51] who described these lesions as "yellowish material that looks like soft drusen arranged in an ill-defined network of broad, interlacing ribbons". The authors also highlighted their more advanced and severe nature compared to soft drusen. Arnold et al. [52], in 1995, coined the term "reticular pseudodrusen," merging both names. They also noted that most patients with these lesions eventually developed MNV [52].

**Fig. 3:**
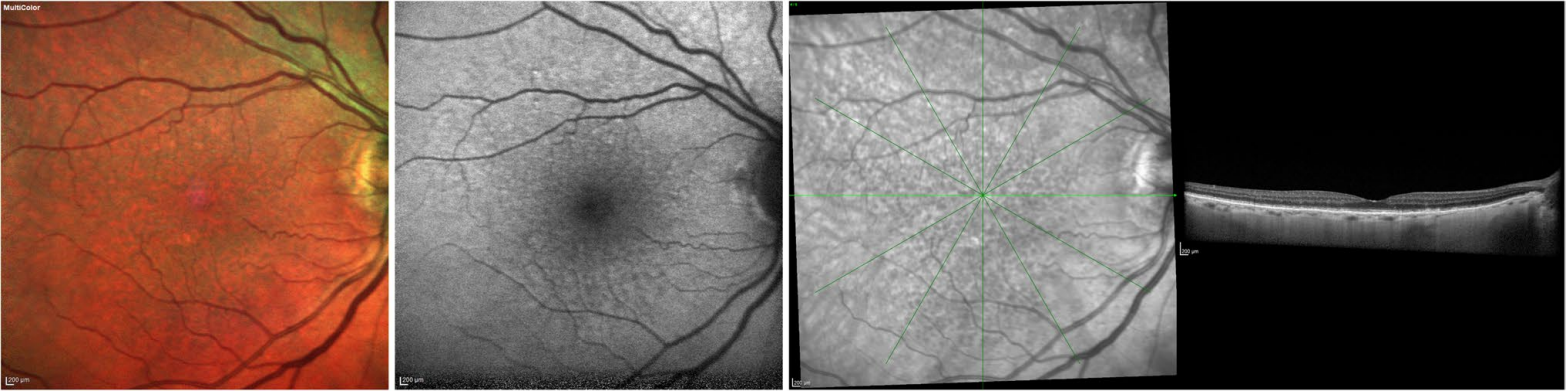
Multimodal imaging from a patient with reticular pseudodrusen. Reticular pseudodrusen were discernible in the multicolor image (left) as green spots and in blue fundus autofluorescence (middle image) as hypoautofluorescent spots. In the infrared image and its corresponding structural OCT image (right), hyperreflective material was evident over the retinal pigment epithelium, aligning with the hyporeflective spots observed in the infrared image

Reticular pseudodrusen were described using structural OCT images as solid hyperreflective material situated in the subretinal space, positioned between the RPE and photoreceptors (Fig. 3). Given this, these lesions are also recognized as subretinal drusenoid deposits (SDDs) [53]. Their progression using structural OCT reveals three discernible stages: (i) initial phase displays diffuse deposition of hyperreflective granular material between the RPE and photoreceptors; (ii) subsequent stages show an escalation in the deposition of granular material, thus modifying the outer retinal profile; (iii) as the process advances, the material takes on a conical shape, breaching the boundary of the photoreceptors [53]. Successively, a fourth stage in the evolution of SDDs has been recognized: the regression of lesions due to the reabsorption of the material [54]. In the presence of SDDs, outer retinal atrophy is a common finding in the evolution and regression of these lesions [55].

Another categorization was proposed by Suzuki and colleagues [56]. In detail, dot SDDs manifest as dot-like clusters and represent the most common form of SDDs. On OCT images, these SSDs may be visualized as accumulations of cone-shaped, pointed subretinal material extending beyond the boundary of the photoreceptors. Ribbon (i.e., or reticular) SSDs, on the other hand, constitute subretinal accumulations of confluent material arranged in a ribbon or band-like configuration, often exhibiting rounded elevations. These formations are commonly located in the perifoveal region and adopt a reticular pattern. Finally, mid-peripheral (i.e., or confluent) SSDs are small, confluent, rounded lesions primarily found beyond the vascular arcades in the mid-peripheral regions.

The presence of RPD/SDDs is a recognized risk factor for the progression to late AMD, including GA [57]. This was initially suggested by Zhou and colleagues, [58] who performed a post-hoc analysis within the comparison of AMD Treatments Trials (CATT). In the latter study, the presence and type (dot, reticular, or confluent) of baseline pseudodrusen were assessed using CFP, green channel, and blue channel; red-free images; and fluorescein angiography (FA). In the study mentioned, pseudodrusen are identified as an independent risk factor for the progression of AMD into its late stages. Specifically, the study highlights that dot pseudodrusen are linked to the development of MNV (i.e., especially type 2 and 3 MNV) [59]. On the contrary, ribbon RPD are more likely associated with the development of GA over time [58].

The association between SDDs visualized on structural OCT and the development of cRORA has been clarified by Hirabayashi and colleagues [37]. As mentioned above, they employed structural OCT to determine the frequency of multiple biomarkers of intermediate AMD and their relationship with the development of cRORA after 2 years. Results from the latter study indicated that the presence of SDDs is a risk for cRORA development within a 2-year period.

### Intraretinal hyperreflective foci

Intraretinal hyperreflective foci (iHRFs) may be detectable on OCT as round lesions with well-defined contours and reflectivity equal to or greater than that of the RPE (Fig. 2). These iHRFs could exist as solitary spots or clustered and can be found either overlying drusen or in the sensory neuroretina without associated drusenoid lesions [47]. They tend to increase over time in number and gradually move from the outer to the inner retina [60]. iHRFs may correspond to areas of hyperpigmentation as seen on CFP images [61]. Previous important studies have postulated that the emergence of these foci might be linked to the migration of RPE cells within the neuroretina. [62–68] or being an expression of inflammatory changes in the drusen and in the inner retina [69–72]. Zhang and Miller [73] have recently provided a combination of imaging, literature, and laboratory evidence supporting the hypothesis that iHRFs represent portions of the RPE that have undergone a pathological transition from epithelial to mesenchymal states: this phenotypic change would reduce drusen-sustaining components production by the RPE, this eventually resulting in drusen collapse and GA development.

Assuming this, several studies employing structural OCT have demonstrated that the presence of intraretinal iHRFs is associated with a greater risk of atrophy development [37, 41, 60, 74–81].

Using structural OCT, Christensbury et al. [60] confirmed that iHRFs are frequently associated with the presence of drusen. More importantly, their presence was associated with a higher occurrence of existing atrophy at the initial assessment. Another critical study was performed by Lei and colleagues, [82] who evaluated the association between different OCT biomarkers, including drusen volume, hyporeflective foci within drusen, SDDs, and HRFs. Their findings confirmed that HRFs confer the higher odds ratio for disease progression [82]. Nassisi and coworkers [40] also produced an essential piece of work confirming these data, studying the relationship between various spectral domain OCT (SD-OCT) biomarkers and progression to cRORA in the fellow iAMD eyes of patients enrolled in the HARBOR study. Among the identified OCT risk factors, the presence of iHRFs was a relevant risk factor for the development of cRORA, yet no correlation was observed regarding the development of MNV [40]. Different groups also aimed to provide quantitative data on the intraretinal distribution of hyperreflective foci relying on their hyperreflectivity properties on B-scans and *en face* images [40, 74, 78, 79, 83]. These reports showed that the amount, area, and volume of iHRF are all harbingers of atrophy formation [40, 74, 78, 79, 83].

### Basal laminar deposits

Basal laminar deposits (BLamD) are commonly found in association with AMD.

They are characterized histologically by a thickened extracellular matrix between the RPE’s plasma membrane and the basal lamina below, potentially replacing or incorporating basal RPE infoldings [84]. These deposits mainly comprise basement membrane proteins and elongated collagen structures [84]. It is important to distinguish BLamD and basal linear deposits (BLinD), the latter located between the RPE basement membrane and inner collagenous layer of Bruch’s membrane [85].

BLamD develop due to excessive basement membrane secretion from the RPE and can be either physiologically associated with aging (i.e., patchily deposits in normal retinas), or pathological. Among those, two types are recognized [86]:

- Early BLamD: These involve the laminar accumulation of extracellular material between the basal lamina of the RPE and its basement membrane, not visible during a fundus examination, yet indicating early RPE damage.
- Late BLamD: These appear as a delayed accumulation of material within early BLamD, potentially leading to pigmentary changes on the fundus. They represent a sign of advanced RPE dysfunction and incipient atrophy and may be associated with reduced visual acuity.

BLamD determine a separation between the RPE and BM, which can be observed on structural OCT B-scans as a low-lying RPE elevation referred to as a “double layer sign” (DLS). This OCT feature is defined by a thick, highly reflective, and uniform layer, consisting of the RPE, associated with another underlying thin, highly reflective band representing BM [84, 87]. Since type 1 MNV can also appear as DLS on OCT B-scans, efforts have been made to confidently distinguish between neovascular and BLamD-associated DLS. Hirabayashi et al. [37] proposed a classification of DLS lesions into two different types according to their thickness (thin vs thick DLS). The thick DLS differs from the thin DLS since it encompasses more than one layer of low to medium reflectivity between the RPE and BM. While the thin DLS is thought to be correlated with BlamD, the thick DLS is more indicative of MNV presence [88, 89]. The authors reported that the presence of a thin DLS, but not of a thick DLS, is predictive of cRORA development within 2 years.

This distinction is prognostically important since evidence has also suggested that the presence of MNV without exudation could actually be protective against atrophy formation as neovessels may balance the reduced perfusion from the ischemic choriocapillaris [90–92]. On the other hand, the accumulation of BLamD could impede proper perfusion of the RPE by the choriocapillaris, resulting in RPE dysfunction and death [85].

### Incomplete RPE and outer retinal atrophy

As mentioned earlier, in 2017, the CAM group defined cRORA as a region of hypertransmission of at least 250 μm in diameter with an associated zone of attenuation or disruption of the RPE of at least 250 μm in diameter and the presence of overlying photoreceptor degeneration [5]. Conversely, the term iRORA is specifically used for lesions that possess these three characteristics but are smaller in size than 250 μm [93].

Therefore, the presence of iRORA serves as an evident risk factor for the development of complete atrophy (i.e., cRORA). The CAM Report 4 and various studies affirm that these smaller areas tend to evolve into cRORA over a span of time, which could range from months to years. Recognizing the presence of iRORA at an early stage is crucial due to this progression potential [93, 94].

In a recent study, Corradetti and colleagues [95] performed a retrospective study to assess the time course and risk factors for conversion of iRORA to cRORA in intermediate AMD eyes. In detail, 321 consecutive iAMD eyes were screened for onset of iRORA. Among these, eyes that developed iRORA within the first 24 months were closely monitored for an additional 24 months to observe the transition to cRORA. From the initial 321 participants with intermediate AMD, the study identified 87 new instances of iRORA lesions (occurring in 50 eyes from 42 participants) for the analysis of conversion to cRORA. Importantly, within the subsequent 24 months, a substantial proportion, 81 out of 87 iRORA lesions (93.1%), progressed to cRORA, with the median duration for this transition being 14 months. Through a multivariate binary logistic regression analysis, the study found that specific factors at baseline were linked to an accelerated rate of progression from iRORA to cRORA. Notably, the presence of intraretinal hyperreflective foci and the extrafoveal location of iRORA lesions were identified as factors associated with a swifter progression to cRORA.

It is important to note that detecting iRORA using structural OCT B-scans is not without challenges. First, the ability to detect these lesions is dependent on the device employed [96]. In a recent study, researchers compared two structural SD-OCT devices (i.e., Spectralis and Cirrus OCT) to detect iRORA and cRORA lesions in patients with AMD. These patients were imaged on the same day using both devices. The study revealed that a more significant number of iRORA lesions were detected with the Spectralis OCT compared to the Cirrus OCT. However, no significant difference was observed between the two devices regarding the detection of cRORA lesions. Second, the assessment of iRORA using OCT B-scans may have limitations. Some of these lesions might span across the borders of adjacent scans, causing challenges in their precise assessment. Specifically, it is possible for a lesion identified as iRORA on a B-scan actually to be part of a larger cRORA lesion. This misidentification occurs when the B-scan intersects only a segment of a more extensive lesion, thereby underrepresenting its true size. *En face* imaging, particularly when used in conjunction with B-scan images, offers a complementary perspective by providing a two-dimensional overview of the atrophy. This method can reveal the full extent of a lesion that may appear smaller on B-scan images due to the scan's cross-sectional nature. When a lesion's diameter exceeds 250 µm on *en face* images, it substantiates the classification of cRORA, despite its appearance on horizontal B-scans. This was confirmed in a recent study that evaluated and compared the detection of iRORA and cRORA assessed on OCT B-scans versus persistent choroidal hyperTDs assessed by *en face* choroidal OCT images. The latter study showed that *en face* imaging could detect iRORA lesions with a greatest linear dimension ≥ 250 μm in a nonhorizontal *en face* dimension. This emphasizes that certain iRORA lesions might, in reality, be cRORA lesions [97]. Given that the diagnosis of iRORA and cRORA inherently relies on B-scan imaging, adopting a combined diagnostic strategy that integrates B-scans with *en face* images promises to enhance the precision of evaluations. This refined assessment can be realized through the utilization of either SD- or SS-OCT imaging techniques.

It is notable to distinguish iRORA from another common lesion detectable via structural OCT, termed nascent GA. This OCT lesion includes subsidence of the inner nuclear layer (INL) and outer plexiform layer (OPL), a hyporeflective wedge-shaped band within the Henle fiber layer, often accompanied by RPE disturbance and increased signal hypertransmission into the choroid [98–101]. Nascent GA is also associated with the development of atrophy over time and may be considered as a precursor to the development of drusen-associated cRORA. Wu et al. [102] subsequently investigated the predictability of nascent GA, as defined by OCT B-scan, to develop into the conventional clinical endpoint of GA, as defined by CFP. They included a total of 284 eyes from 142 participants with bilateral large drusen (i.e., > 125 mm in diameter). Over a 3-year follow-up, 12 eyes progressed to GA, with nascent GA being identified in 10 of these 12 eyes before the full development of GA. This discovery aligned with their earlier observations, highlighting that eyes diagnosed with nascent GA had a notably heightened risk of progressing to GA compared to eyes without nascent GA. However, it is important to note that this precursor lesion does not encompass all forms of cRORA associated with various precursor lesions, such as SDDs. Additionally, nascent GA might transform into a type 3 MNV, rather than developing into a region of cRORA [103].

### Persistent choroidal hypertransmission defects

**Fig. 4:**
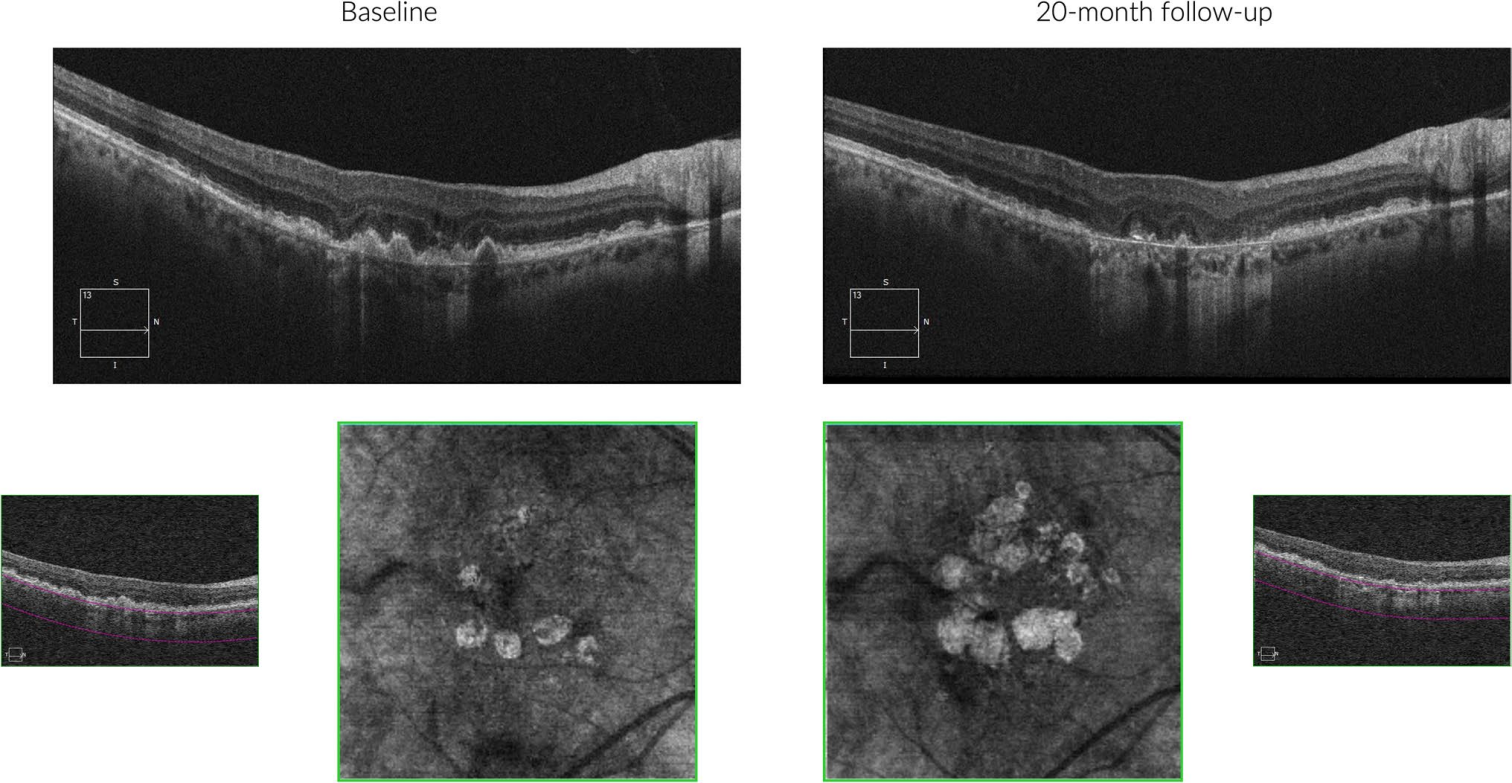
Structural OCT images from a patient with geographic atrophy. A region of cRORA is visible at both baseline and 20-month follow-up visits. The extent of the GA area becomes more readily apparent by opting for a slab beneath the RPE-Bruch’s membrane (bottom), generating an *en face* image that accentuates areas of hypertransmission

HyperTDs are visible on *en face* OCT images as bright areas of increased light transmission in the choroid resulting from the impaired integrity of the RPE (Fig. 4) [104]. HyperTDs could be linked with the existence of a region characterized by cRORA, [105] given that hypertransmission aligns with one of the OCT features of cRORA by its definition. Alternatively, hyperTDs might exist independently of cRORA, as seen in the presence of a nascent GA region. In this latter scenario, hyperTDs could be regarded as a potential risk factor for the eventual development of cRORA over time.

However, the detection of hyperTDs using cross-sectional B-scans may be limited by their apparent transient nature during long-term follow-up [105]. The nonpersistent nature of hyperTDs on B-scans seems to relate to their size and could stem from the transient emergence of tiny areas of hypertransmission or the possibility of missing them during follow-up due to variations in OCT B-scan placement when aligning images with previous visits. To enhance the tracking of small hyperTDs from one visit to another, a potential strategy could involve utilizing *en face* images with closer intervals between B-scans. Subsequently, this OCT *en face* approach employing a slab with segmentation boundaries positioned 64 µm to 400 µm beneath the Bruch's membrane has been largely used to examine the emergence and advancement of GA [105–107]. Previous studies have demonstrated that although some small hyperTDs were transient, larger hyperTD foci seemed to persist, suggesting that a parameter like the greatest linear dimension threshold for HyperTD measured on *en face* images could define persistent lesions more predictive of disease progression. The latter aspect was demonstrated by Shi and colleagues [104] who demonstrated that those hyperTD lesions identified on *en face* sub-RPE images with a greatest linear dimension superior to 250 μm were more likely to be persistent.

A successive study by Laiginhas and collaborators evaluated whether persistent hyperTDs can be considered a biomarker for future GA formation [108]. They analyzed 190 eyes with intermediate AMD over an average follow-up period of 31.0 ± 13.2 months. Initially, 31 eyes (16%) had at least one hyperTD ≥ 250 µm; among these, 13 eyes (42%) progressed to GA. Among the eyes lacking a hyperTD ≥ 250 µm initially, 42 eyes (26%) developed such hyperTDs during follow-up, and 11 eyes (7%) progressed to GA. At the latest follow-up, 25 eyes (13%) had progressed to GA, and among these, a prior hyperTD ≥ 250 µm was detected in 24 eyes before GA formation. A time-dependent Cox-survival regression analysis estimated an 80-fold increased risk (95% CI, 10.7–614, P < 0.001) of developing GA once a hyperTD ≥ 250 µm appeared. These findings collectively suggest that hyperTDs larger than 250 µm, which often persist, represent an independent risk factor for the development of cRORA. In addition, as persistent hyperTDs undergo growth and evolution, they invariably progress to correspond with cRORA lesions as identified on OCT B-scans, and manifest as GA on color fundus imaging. Consequently, the expansion of persistent hyperTDs and the factors contributing to their enlargement are analogous to those affecting GA progression. In particular, using *en face* OCT the growth rate of hyperTDs was measured to be approximately 0.220 mm per year [38].

## Risk factors for the progression of GA

Structural OCT and OCTA may be employed to predict the enlargement of GA over time. In the presence of a region of GA (i.e., cRORA on structural OCT), assessing the border around the RPE atrophy becomes crucial. The imaging characteristics in this area could offer insights into the potential rate of enlargement over time.

### Retinal pigment epithelium to Bruch’s membrane distance

Histopathological studies have revealed a significant thickening of the RPE/BM complex around the areas of atrophy, which appears to be supported by BLamD accumulation. In detail, Sura et al. [84] displayed that in normal retinas, iAMD eyes, and GA regions, Bruch's membrane maintains a consistent thickness (4.2–4.4 µm), while the RPE to BM complex thickness was increased around GA (normal = 13.7 µm, GA = 17.4 µm). These findings suggested that the thickness of the RPE-BM complex is driven by BLamD accumulation around GA areas [84].

Recent studies have associated the thickness of the RPE-BM complex with the progression of GA. Chu et al. [109] examined the RPE-BM distance in a region immediately surrounding the GA area using *en face* OCT images in 38 eyes. Their findings indicated that a thicker distance correlated positively with faster atrophy progression. Similarly, Fleckenstein et al. [110] reported a separation within the RPE-BM complex in eyes displaying rapid GA progression. Finally, no significant correlations were observed between RPE-BM distance and impairment in CC flow within the GA border, suggesting that they should be regarded as independent risk factors [28, 84].

### Outer retinal layer thickness and photoreceptor abnormalities

Multiple pieces of evidence suggest that changes in the outer retinal layers may exist in areas beyond the atrophic region, potentially occurring before the onset of RPE atrophy [111–114]. 

Zhang and colleagues [115] conducted a retrospective study to establish whether the thickness of the outer retinal layer (ORL) surrounding GA could function as a clinical indicator for predicting the annual enlargement rate of GA. They analyzed 38 eyes from 27 participants and discovered that a thinner ORL immediately surrounding GA areas was associated with faster lesion growth, indicating a negative correlation. Furthermore, their findings revealed that together, the RPE to BM distance and ORL thickness could predict approximately 62% of the annual progression of GA. However, when examining ORL thickness alone, it contributed only 6% to this prediction. These results suggest that these factors are not entirely independent risk factors and underscore a strong connection between the loss of photoreceptors and the formation of BLamD [115].

Considering the role of ORL and photoreceptor condition as influential factors in the progression of GA, past studies endeavored to employ deep learning techniques for the automated assessment of ORL and photoreceptor thickness [116, 117]. Through deep learning methods, it was observed that the reduction in photoreceptor loss and thinning could be achieved via intravitreal complement C3 inhibition [118].

### Choriocapillaris perfusion

The CC consists of fenestrated capillaries and plays a key role in normal retinal function by facilitating the passage of oxygen and nutrients to both the RPE and photoreceptors [119].

Several histological ex vivo studies have revealed choroidal changes in early/intermediate AMD that may eventually precede the loss of photoreceptors and RPE [120–123].

McLeod et al. [124] analyzed the postmortem choroids from 11 subjects including 3 age-matched controls, 5 GA subjects, and 3 neovascular AMD subjects. The researchers observed that despite evident loss of RPE in certain areas of GA, the CC could remain intact. Based on these observations, the authors concluded that the initial insult in GA likely occurs at the level of the RPE, with subsequent degeneration of the CC. Following this, Seddon and colleagues [125] examined postmortem choroids from 36 subjects, consisting of 4 age-matched controls and 32 individuals with various stages of AMD, including 5 subjects with GA. This subsequent study confirmed a significant reduction in the CC in eyes with GA, predominantly localized to regions of RPE atrophy. Within the GA region, some CC vessels may persist, albeit with a notable reduction in diameter, implying both morphological and functional alterations in these surviving vessels. Lastly, Edwards et al. [126] conducted a recent analysis on postmortem choroids from eight subjects, which included five age-matched controls and three siblings with GA. Notably, imaging data were available for the donors prior to death, enabling clinicohistopathologic correlations. In GA eyes, they observed severe CC dropout, with a pattern and size directly corresponding to regions of RPE atrophy. Surviving CC vessels in these regions appeared constricted. Conversely, in areas where RPE was present, the CC vessels were comparable in number and macroscopic appearance to those in healthy age-matched controls. However, fewer fenestrations per capillary were observed compared to aged control CC, particularly evident in border regions. This suggests that while the CC may appear normal at the border of GA, some functional changes may already be occurring in these vessels in this region. Consistently, Li and colleagues observed a progressive decline in CC density from non-atrophic to atrophic regions, encompassing the margins of the GA [127]. Hence, despite uncertainties, a proposed theory posits that vascular alterations predominantly impact the CC in GA as a result of degenerative, inflammatory, or genetic processes. This sequence subsequently induces ischemia and compromises the RPE [120, 127, 128].

Using OCTA in vivo in GA patients, Nassisi et al. [129] appeared to validate the latter findings, showing that the CC flow is highly reduced in areas immediately adjacent to atrophy, where the RPE remains structurally intact. Given the importance of the CC in AMD and GA, as mentioned earlier, several studies have sought to investigate whether OCTA metrics may predict the progression of GA over time. Various studies have consistently shown that reduced blood flow in the CC in the region immediately surrounding the GA border stands as a significant risk factor contributing to the accelerated expansion of GA over time [30, 130, 131].

An important study by Thulliez et al. [29] sought to investigate the association between GA enlargement rate and CC perfusion in the region immediately surrounding the GA border, as well as in the total scan area. Surprisingly, the CC perfusion in the total scan area was better correlated with the GA enlargement rate than the CC perfusion in the region immediately surrounding the GA border. The probable reason for this result is that the natural age-related rise in CC hypoperfusion within the central macula [132] might complicate the links between the expansion rates of GA and CC flow deficits. Conversely, areas farther from the edges of GA are less influenced by typical age-related changes, making them more indicative of CC changes linked to the severity of AMD. A successive and similar study further confirmed the latter findings [133].

Structural OCT also plays a role in evaluating the choroid to predict the progression of GA over time. This imaging modality reflects the ratio between choroidal vascular and stromal components. This metric has shown a correlation with GA expansion rates over time, where lower values (i.e., indicating a reduced vascular component) were linked to a swifter rate of atrophy enlargement at one and two years [134].

### Involvement of the foveal region in patients with extrafoveal GA

Given that GA eyes treated with pegcetacoplan exhibit notably slower progression toward the fovea, [135] it becomes clinically pertinent to identify risk factors associated with such progression. In a prior investigation conducted by our group, [136] we examined eyes presenting with extrafoveal GA at baseline to evaluate the presence of risk factors for foveal involvement over a 24-month period. This study highlighted two significant baseline risk factors: thinning of the outer retina in the foveal region and the presence of baseline large drusen or BLamD in the foveal region.

## Conclusions

GA commonly complicates AMD, often leading to a more unfavorable prognosis among affected patients. With the emergence of new treatment approaches for GA, there is an even greater demand for timely diagnosis and accurate monitoring of patients with this condition. Employing structural OCT and OCTA becomes imperative in identifying specific biomarkers linked to the onset and advancement of GA. Understanding the significance of certain structural OCT features such as drusen volume, specific drusen characteristics, and the presence of various elements like subretinal drusenoid deposits, hyperreflective intraretinal foci, BLamD, iRORA, and persistent choroidal hyperTDs is crucial. These features are linked to an increased risk of progressing to GA. In cases where GA is already present, exploring the GA border via structural OCT and OCTA is recommended, as characteristics in this area are correlated with GA expansion over time according to existing literature. It is important to note that ongoing studies focused on identifying imaging biomarkers in AMD and GA will likely lead to modifications and expansions in this list as our understanding evolves. Moreover, the integration of artificial intelligence with these biomarkers holds significant promise for enhancing their clinical utility. Specifically, artificial intelligence applied to OCT devices has the potential to provide predictive probabilities of GA development over time. This advancement could enable adjustments to follow-up intervals, allowing for prompt initiation of treatment upon GA detection.
